# Innovative strategies to predict and prevent the risk for malnutrition in child, adolescent, and young adult cancer survivors

**DOI:** 10.3389/fnut.2023.1332881

**Published:** 2023-12-21

**Authors:** Fiorentina Guida, Laura Andreozzi, Daniele Zama, Arcangelo Prete, Riccardo Masetti, Marianna Fabi, Marcello Lanari

**Affiliations:** ^1^Paediatric Emergency Unit, Department of Medicine and Surgery, IRCCS Azienda Ospedaliero-Universitaria di Bologna, University of Bologna, Bologna, Italy; ^2^Pediatric Oncology and Hematology Unit "Lalla Seragnoli", Pediatric Unit-IRCCS Azienda Ospedaliero-Universitaria di Bologna, Bologna, Italy

**Keywords:** children cancer survivors, malnutrition, nutritional intervention, artificial intelligence, risk assessment

## Abstract

Children, adolescents, and young adult cancer survivors (CAYAs) constitute a growing population requiring a customized approach to mitigate the incidence of severe complications throughout their lifetimes. During cancer treatment, CAYAs cancer survivors undergo significant disruptions in their nutritional status, elevating the risks of mortality, morbidity, and cardiovascular events. The assessment of nutritional status during cancer treatment involves anthropometric and dietary evaluations, emphasizing the necessity for regular assessments and the timely identification of risk factors. Proactive nutritional interventions, addressing both undernutrition and overnutrition, should be tailored to specific age groups and incorporate a family-centered approach. Despite encouraging interventions, a notable evidence gap persists. The goal of this review is to comprehensively examine the existing evidence on potential nutritional interventions for CAYAs cancer survivors. We explore the evidence so far collected on the nutritional intervention strategies elaborated for CAYAs cancer survivors that should target both undernutrition and overnutrition, being age-specific and involving a family-based approach. Furthermore, we suggest harnessing artificial intelligence (AI) to anticipate and prevent malnutrition in CAYAs cancer survivors, contributing to the identification of novel risk factors and promoting proactive, personalized healthcare.

## 1 Introduction

Children, adolescents, and young adults (CAYAs) cancer survivors cancer survivors are a growing population that need a tailored approach to reduce the incidence of severe complications in a life-long period. The development of effective nutritional programs is inherently difficult in CAYAs due to low compliance with dietary recommendations from healthcare providers and the difficulty in adhering to these indications over time (1).

The effectiveness and feasibility of an appropriate nutritional intervention in cancer survivors are even more challenging, but it represents a substantial strategy that should consistently proceed from diagnosis to follow-up. Indeed, nutritional status impairment is a common event in children diagnosed with cancer and a recognized risk factor for increased morbidity and mortality (2).

According to the definition of the World Health Organization, both undernutrition and overnutrition can be considered as different expression of malnutrition (3) and in CAYAs cancer survivors this definition perfectly matches.

During cancer treatment, well-known adverse effects of cancer therapies (i.e., emesis, mucositis, diarrhea) and the alteration in metabolic status and inflammatory response (4) expose these children to undernutrition. The burden of undernutrition could have an impact on overall survival and quality of life, affecting the response of children to cancer treatment and their long-term prognosis (2).

After completing cancer treatment, CAYAs cancer survivors can be more exposed to overnutrition, due to a combination of poor dietary habits and a sedentary lifestyle with profound metabolic alteration secondary to the cancer treatment itself (5, 6). Overweight and obesity increases cardiovascular risk and mortality and are related to greater fatigue, reduced physical activity and depression, deeply affecting the quality of life (7, 8).

Managing malnutrition in CAYAs cancer survivors can be challenging for many reasons.

Firstly, CAYAs cancer survivors experiment high prevalence of bad lifestyles habits that expose them to a greater risk for comorbidity and cardiovascular diseases that persist to the adulthood (9). In a recent cohort, one out of two did not meet physical activity guidelines, two out of three were overweight or obese, one out of five were current smokers and one out of ten consumed high amounts of alcohol (10). Most cancer survivors tend to adopt negative eating behaviors, characterized by excessive salt intake (≥ 10 g per day) reduced consumption of fruits and vegetables, and a high intake of saturated fatty acids. Additionally, cancer survivors appear to struggle in achieving the guidelines’ recommended levels for vitamin D, calcium, and saturated fats (11).

In addition to these unhealthy eating habits, CAYAs cancer survivors do not engage in regular physical activity, exhibiting diminished motor performance compared to their peers at the end of the acute treatment phase (12), with an even more pronounced mobility limitation two years after completing the therapies (13–16). The benefits of regular physical activity in cancer survivors are not limited to countering the frequent weight gain these patients often experience. An interesting study has shown that patients diagnosed with Hodgkin’s lymphoma who engaged in regular vigorous physical activity at the end of treatment had a lower cumulative incidence of cardiovascular events compared to similar patients who were physically inactive (17).

Secondly, there are few available evidence on which nutritional recommendations can be used for children diagnosed with cancer (18). Dietary recommendations so far collected can be inherited from adults’ guidelines (18, 19) as practical advice for healthy lifestyles that differ only slightly from that available for general population (20). Finally, once established, bad dietary habits and inadequate exercise levels are difficult to reverse (21).

CAYAs cancer survivors and their families experienced significant and distressing nutrition challenges and perceived as reassuring the chance to receive a nutritional support (22). However, only one study has proven the efficacy of a one-year nutritional intervention program in improving diet quality of children diagnosed with cancer (23).

Therefore, the risk for malnutrition, the difficulties in reverting bad lifestyles habits and the burden of this conditions on the overall survival and quality of life (24) highlight the importance of developing and validating prevention programs. Since its implementation in the medical sciences, the AI has proven to be a valuable tool in the field of biomedical sciences, and particularly in nutrition and food science (25, 26), in dietary pattern analysis (27), and in personalized nutrition (28, 29), from the evaluation of molecular differences in food composition (30, 31) to the optimization of the production of specific dietary compounds (26, 32).

Finally, AI has already proven its practical utility in childhood cancer survivors, from the early detection of cardiotoxicity to the achievement of a tailored therapeutic approach (33). Another pivotal role that could be played by AI is the prediction of risk factors involved in numerous chronic diseases, including cancers, metabolic syndrome and cardiovascular (34).

After conducting a brief review of the available evidence regarding potential nutritional interventions in CAYAs cancer survivors, we explored the potentiality to harness AI to predict the risk of malnutrition and to develop nutritional intervention strategies in this fragile population.

## 2 Methods

A systematic literature search was conducted using electronic databases, including PubMed. The search terms included “cancer survivors” and variations of “nutritional status,” “dietary interventions,” “recommended dietary allowances” and “diet therapy.” The search was limited to children, adolescent, and young adults and to articles published in the last five years. Original research studies and reviews on the relevance to nutritional interventions for CAYAs cancer survivors were included.

## 3 Results

A total of fifteen studies were identified, twelve of which were included in the mini review.

The selected original researched and reviewers aimed to assess the nutritional status and dietary characteristics of CAYAs cancer survivors, to recognize potential risk factors for malnutrition, and to define of the peculiarity and advantage of a nutritional intervention, as depicted in Table 1.

**Table 1:** Principal aims of the studies included in the mini-review.

Authors, year	Aim/purpose		
	Assessment of the nutritional status and dietary charachteristics	Potential risk factors	Nutritional intervention
Wiernikowski et al., 2023 (35)	Yes	No	No
Van der Haak et al., 2021 (36)	Yes	Yes	No
Muscaritoli et al., 2019 (37)	Yes	No	No
Delvin et al., 2019 (38)	Yes	No	No
Belle et al., 2019 (39)	Yes	No	No
Raber et al., 2020 (40)	Yes	No	Yes
Wu et al., 2022 (24)	Yes	No	Yes
Podpeskar et al., 2021 (41)	Yes	No	Yes
O’callaghan et al., 2022 (42)	Yes	No	Yes
Lan et al., 2023 (43)	Yes	No	Yes
Raber et al., 2019 (44)	Yes	No	Yes
Bèrard et al., 2020 (45)	Yes	Yes	No

### 3.1 Why it’s important to address malnutrition: assessing nutritional status in CAYAs cancer survivors

Malnutrition seems to be associated with an overall worse prognosis, exposing the patient to an increased overall and cancer-specific mortality (46). Besides, the increased incidence of overweight and obesity during the follow-up of cancer survivors results in a further elevation of cardiovascular risk, increasing the likelihood of chronic conditions such as arterial hypertension, dyslipidemia, and metabolic syndrome (47).

CAYAs cancer survivors are at higher risk for developing cardiovascular events experiencing symptomatic and asymptomatic left ventricular disfunction even several years after cancer treatment (47). The overall incidence of cardiotoxicity is difficult to establish, varying from 16% to 50% of children exposed to well-known cardiotoxic drug as anthracyclines (48). Interestingly, the incidence of cardiomyopathy and its severity are higher in children with two or more modifiable cardiovascular risk factors (49). In general population a youth onset obesity has been associated to accelerated and early vascular aging, exposing these children to higher incidence of cardiometabolic disease across the lifespan (50). The development of atherosclerotic cardiovascular disease is influenced by a socio-ecological framework in which low socioeconomic status, food insecurity, and early childhood adversity are well recognized factors (51). The same social features have been described in CAYAs cancer survivors and their families (9) thus strengthening the role of atherosclerosis as an important cardiovascular risk factors in this population.

Furthermore, dyslipidemia and obesity are well-known identified modifiable cardiovascular risk factors also in children exposed to cancer treatment in which prevention strategies can dramatically reduce the burden of cardiovascular mortality in adulthood (51).

Among a large cohort of adults who had overcome childhood cancer, those who consistently followed healthier dietary patterns exhibited a decreased risk of cardiovascular diseases, including individuals with elevated inherent cardiovascular risk (43).

Therefore, the evaluation of nutritional status at the diagnosis, during the cancer treatment and the long-term follow-up could be a relevant element of the multidisciplinary assessment of cancer survivors to continuously investigate.

The first assessment of nutritional status should be performed at diagnosis. It should include an accurate investigation of the anthropometric parameters involving standard parameters (weight, height, body-mass index, BMI) and advanced measures (mid-upper circumference, body composition assessment) (52, 53). Indeed, mid-upper-arm circumference has been proven to correlate with lean body mass and adipose tissue, being associated to cardiovascular risk in children (54) and in CAYAs cancer survivors this measure could be even more useful to correct a false evaluation of overnutrition in children with oedema or large tumors (55). Another relevant component of nutritional assessment should consider patient’s dietary history (food aversion, allergies or food intolerance, diary assumption of macronutrients and micronutrients) and laboratoristic alterations (liver and renal function panel, lipid panel, glucose monitoring) (56).

Particularly, to estimate lipid alteration (hypertriglyceridemia and/or dyslipidemia) could impact the pre-treatment cardiovascular risk assessment (56), potentially contributing, in part and in theory, to therapeutic choices. In children treated for acute lymphoblastic leukemia that need to receive a coadministration of steroids and L-asparaginase it could be t might be beneficial to contemplate a low-fat diet (53). This is supported by evidence from a murine model, where a low-fat diet demonstrated enhanced survival and increased sensitivity to chemotherapy (57). In turn, the administration of steroids could be complicated by hyperglycemia that can promote and worsen the incidence of overweight (58). Hyperglycemia and dyslipidemia expose children with cancer diagnosis to higher risk for infectious disease and sepsis that are worsened by cancer treatment related immunodepression and catabolic state (53). However, it could be challenging for these children to adhere to a low-calorie diet, considering the appetite fluctuation associated with high dosage of steroids (25).

From the analysis of dietary intake of children diagnosed with acute lymphoblastic leukemia (59), diary total calories intake exceeded the recommended values with a greater incidence of overweight or obesity up to 27% (59). Furthermore, the same population experienced lower dietary intake of calcium and vitamin D that can further complicate the adequate bone development which is already disrupted by some cancer treatment as steroids (59, 60).

### 3.2 Risk factors for malnutrition

Undernutrition is related to highly emetogenic regimens, drug therapy associated with gastrointestinal complication (constipation, diarrhea, loss of appetite, mucositis, or enterocolitis), and radiotherapy of the upper gastrointestinal. Similarly, surgical procedures that involve gastrointestinal tract could be associated to prolonged ileus or short gut syndrome that can compromise the absorption of nutrients and mucositis secondary to stem cell transplantation can determine the same inauspicious outcome (53). Some malignancies have been associated with high occurrence of gastrointestinal complication such as Burkitt’s lymphoma, osteosarcoma, and central nervous system tumors (53) Furthermore, patients with acute lymphoblastic leukemia, lymphoblastic lymphoma, and acute myeloid leukemia had proven to experience significative loss of weight during cancer treatment (61). The evidence of malnutrition or cachexia before the start of cancer treatment, together with the need for prolonged parenteral nutrition are other high-risk factor for developing undernutrition during cancer treatment. Finally, some socio-demographical factors have been related to an increased incidence for malnutrition, such as a young age at diagnosis and a low socio-economic status that limit the availability of nutrients (53).

On the other hand, overweight or obesity during and after cancer treatment are related to diagnosis of acute lymphoblastic leukemia (10, 61) and the evidence of excessive body weight at diagnosis (53). Also the administration of high and prolonged dosage of corticosteroids as well as other cytoreductive agents (i.e., L-asparaginase) can induce metabolic alteration that cause an increased adipose tissue deposition and a reduction in muscular tissue (62). Furthermore, neurotoxic and cardiotoxic effects of cancer treatment can reduce exercise tolerance and induce ventricular disfunction, bringing to mobility limitations (10) that exacerbate the risk for overweight and obesity (73). Moreover, a diagnosis of acute lymphoblastic leukemia (45), lymphoblastic lymphoma and acute myeloid leukemia are more likely to develop overweight and obesity during survivorship (36). Lastly, since patients and physicians hold differing perspectives on cancer-related nutrition, enhancing physician focus on nutrition and providing patients with information to optimize their dietary choices is crucial for improving overall quality of life (37).

### 3.3 Nutritional interventions

In regard to counteract malnutrition in CAYAs cancer survivors, it is essential to elaborate a proactive nutritional intervention focused on the aim to prevent undernutrition and overnutrition rather than to revert them (41).

Malnutrition resulting from dietary changes or weight loss in cancer patients may heighten the risk of infection and other adverse effects associated with chemotherapy. Temporary or mild protein-calorie malnutrition may have a lesser impact on antineoplastic pharmacokinetic. More significant nutritional deficits can lead to the loss of adipose tissue, lean tissue, and essential nutrients with increased extracellular fluid volume. Malnutrition could alter organ function, with reductions in cardiac output, glomerular filtration, and hepatic blood flow, negatively impacting oxidative liver functions (35).

As for overnutrition and obesity, a recent Cochrane review analyzed the role of nutritional intervention in reducing the risk for cardiovascular and metabolic disease specifically focused on survivors of childhood cancer (40). The studies included in the analysis varied for ages of the enrolled participants, the timing of intervention and the primary outcomes considered but showed that CAYAs cancer survivors consume a high intake of free sugars, processed and refined foods, sodium and few fruits and vegetables (45, 52). Children survived to acute lymphoblastic leukemia had a week adherence to dietary recommendations with more than half of participants’ daily energy intake that was provided by ultra-processed foods. Furthermore, a weak adherence to diet is associated with alteration in several cardiometabolic outcomes (hypercholesterolemia and hypertriglyceridemia, insulin resistency, elevated blood pressure). Diet quality also influenced circulating adiponectin levels and TNF-α (45), associated with low-grade inflammation, possibly leading to insulin resistance and diabetes and contributes to the pathogenesis of atherosclerosis (45).

The Mediterranean diet, or DASH (Dietary Approaches to Stop Hypertension), could serve as a valuable model for CAYAs cancer survivors and their families to follow (40). Considering the tendency of CAYAs cancer survivors to consume high amounts of sodium (39), saturated fats, and processed foods, adhering to the recommendations of this diet regarding the limitation of these foods could be an additional suggestion for families.

Lastly, as a result of the alteration of the intestinal microbiota that occurs during chemotherapy, promoting the adoption of nutritional strategies that can preserve or restore adequate intestinal bacterial flora is another goal of nutritional intervention. Particularly, N3 polyunsaturated fatty acids play a crucial role in maintaining gut health and modifying the gut microbiome (41). In CAYAs cancer survivors, they exert various beneficial effects on the immune system, various metabolic pathways, and proliferation processes. The administration of docosahexaenoic acid and eicosapentaenoic acid appears to be a relatively non-toxic form of supportive therapy but the existing evidence does not permit the development of recommendations on this issue (41).

In addition to providing dietary recommendation, the physician should be able to suggest practical and easily implementable nutritional strategies, considering that the family’s adherence to diet is as important as the relevance of the nutritional recommendation. Suggesting a weekly meal planning with variate recipes can ameliorate family’s compliance to dietary recommendation. An interesting study conducted on CAYAs cancer survivors, and their families revealed that meal preparation can be a source of stress and frustration for both the families and the CAYAs cancer survivors. After surviving cancer, families tend to allow the child to eat whatever they desire, potentially heightening the risk of unhealthy behaviors. This approach also resulted in a loss of control for the parents that perceived protectiveness and concern over their child and its eating habits (40). Observing the meal preparation habit of CAYAs cancer survivors’ families, the use of well prepared and adequate leftovers (balanced meals cooked to one evening that are used for school lunches or as a component in a subsequent meal) can promote healthy behaviors. Another key element in meal planning is understanding the preferences of CAYAs cancer survivors and their families. A strong aversion or lack of familiarity with specific foods, textures, and flavors can pose a significant challenge to dietary changes within families. A careful consideration of these preferences is crucial when crafting nutritional interventions targeting meal preparation behaviors, particularly given the potential alterations in taste and smell resulting from cancer treatments (44). Furthermore, encouraging home-cooking and meal sharing with the whole family has proven to be a reliable strategy to arouse CAYAs cancer survivors and their families’ adherence to a healthy diet (40). Lastly, CAYAs cancer survivors tends to express the need for a specific nutritional advice and dietetic referral, often struggling with weight gain and inadequate dietary habits during survivorship (42).

However, given the limited availability of research and the diversity in methodologies across the examined studies, definitive conclusions regarding the efficacy of nutritional interventions for childhood cancer survivors cannot be established (52).

Nevertheless, it has still been possible to make some considerations regarding the characteristics that a nutritional intervention should possess to be adequate. A suitable nutritional intervention should be age-specific, varying from toddlers to adolescents who are at greater risk for unhealthy behaviors. It should target any underlying conditions and high-risk groups, attempting to reduce the risk for undernutrition during cancer treatment and simultaneously lower the incidence of metabolic syndrome in the long-term follow-up. A diet with low glycemic index, high protein assumption, and greater intake of fibers as well as a Mediterranean-style diet have proven to be effective in reducing risk factors related to metabolic syndrome (61, 62). Besides, to be effective an adequate nutritional intervention should involve a family-based approach aimed to deeply change lifestyle habits (63). Indeed, being able to propose practical strategies to the family unit of CAYAs cancer survivors could be a key step to the formulation of a tailored nutritional intervention. Frequently, the physician suggests the implementation of healthy eating habits, advising the adoption of a diverse diet high in fiber and oligoelements (38), avoiding an imbalance towards high-carbohydrate or high-fat foods or highly processed foods. However, these recommendations are often vague and lack practical guidance, sometimes leaving families disoriented rather than assisted. To elaborate a practical list of recipes and foods that can be variously combined, which the family can easily prepare and organize periodically (such as weekly) could be a more effective strategy. In this way, the nutritional intervention would involve the practical suggestion of a list of recipes to be combined into a meal planning, ideally based on the patient’s preferences.

Given the complexity of managing cancer survivors providing them a suitable nutritional intervention, the gap in the evidence remains considerably wide. Furthermore, blending the nutritional requirements of a well-balanced diet with the preferences of the survivor and their family, while ensuring long-term adherence, is likewise very challenging. But, what if artificial intelligence (AI) could fill the gap?

### 3.4 The role of artificial intelligence in predicting and preventing the risk for malnutrition: future perspectives

We aimed to explore the potential role of AI in predicting the risk for malnutrition and as a personalized nutritional assistant for CAYAs cancer survivors and their families.

In pediatric population, AI has demonstrated accuracy in disease prediction models, from the management of chronic conditions as diabetes (64) and metabolic kidney disease (65) to the elaboration of treatment planning for congenital heart disease (66). Furthermore, an incisive example of the potential role of AI in risk prediction model has been demonstrated in myocardial infarction, in which AI exhibited superior accuracy compared to traditional models, offering real-time performance improvements that enhance the prognosis and cost-effectiveness of this condition (67).

Therefore, AI could be implemented in risk prediction strategies to identify children at higher risk for undernutrition and/or overnutrition during cancer therapies and in the long-term follow-up. By analyzing extensive patient data, including medical records, treatment history, nutritional details, and lifestyle factors, AI can process and interpret vast datasets provided by physicians.

Based on these data, AI could build a predictive model for the risk of malnutrition in CAYAs during the antineoplastic treatment and toward the long-term follow-up. In the event of being identified as high risk for malnutrition, the physician could intensively monitor the patient, enabling the prompt and effective implementation of corrective strategies. Lastly, through real-time monitoring, AI can continuously monitor relevant parameters, incorporating real-time data gathered from the patients’ features and alerting healthcare providers when the risk for malnutrition is increasing.

**Figure 1:**
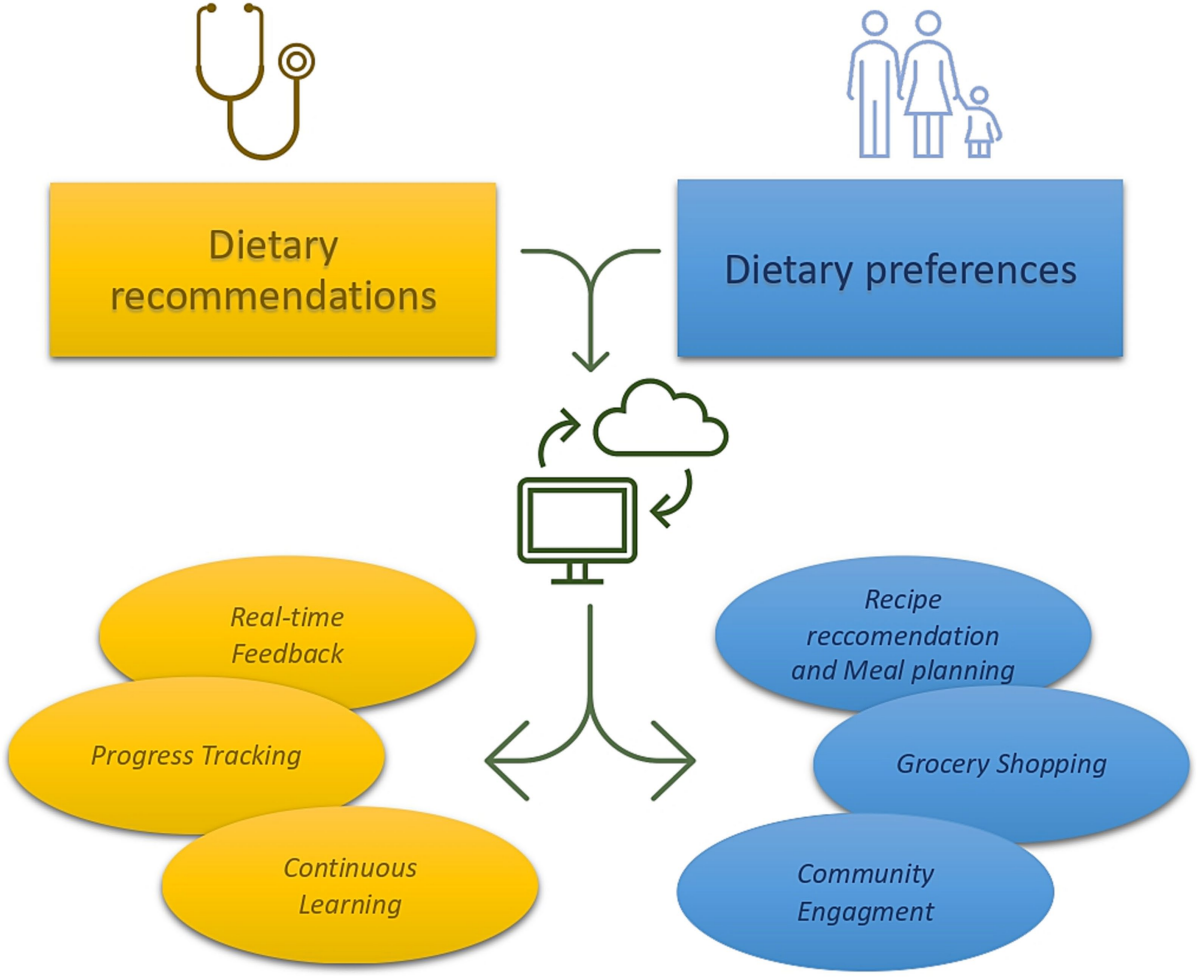
Hypothetical model of nutritional intervention that combines physician’s recommendation, patients’ preferences, and AI intervention. The physician, marked in blue, establishes nutritional guidance based on patient’s age and specific clinical records. These recommendations are then provided to the AI that simultaneously receives information about patients and families’ dietary preferences. AI combines these data offering feedback to both the physician and the patients (recipe recommendation and meal planning). The patients could receive recipe suggestions and meal planning, receiving feedback to nutritional quality of the food bought and consumed through photo analysis (grocery shopping). Lastly, AI could combine information provided by other CAYAs family on a virtual platform (community engagement), thus ameliorate its performance.

Another interesting field of application is based on the role of AI in clinical practice, supporting physicians in providing and modulating dietary characteristics of CAYAs and their families. In the setting of chronic diseases, such as type 2 diabetes, the implementation of AI and mobile supported nutritional intervention exhibited efficacy in improving glucose balance (68). When combined to physician contribute, AI was effective in improving adherence to a healthy lifestyle, reducing body mass index and body fat percentage in a population of overweighted adults (69). In CAYAs cancer survivors, the implementation of AI could help physicians in elaborating a tailored and efficient nutritional intervention, especially in the long-term follow-up. A potential AI or even mobile supported model could involve a continuous cooperation between the physician, the patients, and their families (Figure 1).

Firstly, based on Mediterranean and DASH diet recommendations, the physician could establish the diary caloric amount and the proportion of macronutrients, considering the age and other specific patient’ clinical records.

Secondly, through the implementation of questionaries, the patients could express its own dietary preferences (i.e., vegetarian or vegan, dietary restrictions). Indeed, the *Automated Self-Administered 24-Hours Dietary Assessment Tool* (ASA24) has proven to be compatible with machine learning models and computational methods, providing useful evidence in lactose assumption (70). Other useful questionnaires that have been tested in CAYAs cancer survivors are the *Harvard Service Food Frequency Questionnaire* (HSFFQ) and the *Youth and Adolescent Food Frequency Questionnaire* (YAFFQ) (59). These questionaries contain a list of food and food portion size that can deeply investigate the nutritional habits of children younger than five years-old up to eighteen years old (71, 72). Using these tools, children and their family could provide their nutritional preferences to the AI that could elaborate a nutritional plan through machine learning. Combining patients’ dietary preferences with physician indications, AI could develop a weekly or monthly meal planning that suggests personalized meal options and recipe recommendations based on the patient’s profile.

Through the analysis of the consumed meal, AI could provide bidirectional feedback to the physician and the patients. The physician would be informed about the calories consumed during the observation period and the distribution of macronutrients in meals, being able to make minor adjustments remotely as well. The patient could receive positive or negative feedback based on adherence to clinical guidelines. Furthermore, envisioning the possibility of creating a community across different platforms (i.e., mobile app) could enhance positive reinforcement through the achievement of communal goals. In this regard, allowing families and patients to share their experiences and recipes with other CAYAs cancer survivors could also be beneficial.

Furthermore, through the computational analysis of images, another role of AI could be to identify the nutritional and caloric characteristics of a meal from its photo. A system equipped with such capability could assist CAYAs cancer survivors and their families in identifying the product with the best nutritional properties, directly at the grocery store. In this setting, AI could offer suggestions for healthier alternatives and notify users of unhealthy products, providing real-time feedback on meals consumed and offering suggestion for improvement.

Lastly, through a periodical inclusion of health metrics (weight, height, adherence to dietary goals over time) by the patients, AI could provide real-time feedback also to the physician who can continuously contribute to enhancing the AI’s understanding through reinforcement learning, thereby improving the system over time.

## 4 Conclusion

Managing malnutrition in CAYAs cancer survivors is intricate, given the prevalence of unhealthy lifestyle habits and the paucity8 of evidence-based nutritional recommendations specific to this population. The impact of malnutrition on overall survival and quality of life underscores the need for proactive prevention programs. While nutritional interventions have shown promise, a comprehensive understanding of effective strategies remains elusive, leaving a significant gap in evidence. Recognizing the potential of AI to revolutionize healthcare, we explored the role of AI in predicting and preventing the risk of malnutrition in CAYAs cancer survivors. Through machine learning and deep learning, AI can process vast amounts of patient data, offering predictive models that assess individualized risks based on diverse parameters. Real-time monitoring capabilities enable early intervention, alerting healthcare providers to increasing risks. Additionally, AI has the potential to identify novel risk factors, contributing valuable insights for future research.

On the other hand, the physician, marked in yellow, receives update on health metrics, adherence to nutritional intervention and patients’ dietary habits (progress tracking) at any time (real-time feedback). Finally, providing continuous feedback to the AI (continuous learning), the physisican would contribute to the constant improvement of the system.

## Author contributions

FG: Conceptualization, Investigation, Supervision, Writing – original draft, Writing – review & editing. LA: Investigation, Methodology, Validation, Visualization, Writing – review & editing. DZ: Conceptualization, Supervision, Validation, Visualization, Writing – original draft, Writing – review & editing. AP: Conceptualization, Supervision, Validation, Writing – review & editing. RM: Conceptualization, Supervision, Validation, Visualization, Writing – review & editing. MF: Supervision, Validation, Visualization, Writing – original draft, Writing – review & editing. ML: Supervision, Validation, Visualization, Writing – review & editing.
